# Muscle involvement with pseudohypertrophy in systemic light chain amyloidosis: Case report

**DOI:** 10.1097/MD.0000000000028267

**Published:** 2021-12-23

**Authors:** Mirela Draghici, Andreea Jercan, Sorina Nicoleta Badelita, Ruxandra Maria Irimia, Alexandra Eugenia Bastian, Camelia Dobrea, Monica Popescu, Daniel Coriu

**Affiliations:** a Fundeni Clinical Institute, Bucharest, Romania; b “Carol Davila”University of Medicine and Pharmacy, Bucharest, Romania; c Colentina Clinical Hospital, Bucharest, Romania.

**Keywords:** carfilzomib, case report, light chain amyloidosis (AL), macroglossia, muscle pseudohypertrophy, proteasome inhibitors

## Abstract

**Rationale:**

Muscle pseudohypertrophy is a rare manifestation of light chain amyloidosis (AL) amyloidosis.

**Patient concerns:**

A 63-year-old woman presented with a 2-year history of progressive asthenia, macroglossia, dysphonia, cachexia, hypotension, paresthesia, and lower limb muscle hypertrophy.

**Diagnosis:**

Free serum lambda light chains were increased, and fat pad biopsy demonstrated Congo red-positive deposits. Additionally, electromyography showed a myopathic pattern, whereas muscle biopsy revealed amyloid deposits. A diagnosis of λAL with cardiac, renal, nervous system, and skeletal muscle involvement was established.

**Interventions and outcomes:**

The patient received 3 subsequent lines of therapy over the following 23 months, with very slow hematological remission followed by resolution of organ dysfunction.

**Lessons:**

Despite its rarity, muscle involvement should be considered in patients diagnosed with AL amyloidosis associated with unexplained muscle hypertrophy or weakness associated with macroglossia or elevated troponin T levels in the absence of clear cardiac involvement.

## 1 Introduction

Light chain amyloidosis (AL), also known as primary amyloidosis, is one of the most common subtypes of systemic amyloidosis. In AL amyloidosis, tissues and organs are infiltrated by unstable immunoglobulin light chains, secreted by a clone of malignant bone marrow-residing plasma cells. Commonly affected sites include the peripheral and autonomic nervous system, heart, kidney, liver, and gastrointestinal tract. The symptoms are nonspecific, leading to a delayed diagnosis, when irreversible organ damage is already installed, and the outcome is nearly invariable fatal.^[1]^ Myopathy is a rare manifestation of AL amyloidosis, clinically expressed as muscle weakness, myalgia and very rarely as pseudohypertrophy.^[2,3]^ We describe the case of systemic AL amyloidosis with associated muscle involvement that presented as pseudohypertrophy. The patient gave permission for inclusion in the manuscript.

## 2 Case presentation

A 63-year-old woman was referred to our center in September 2017, following a 2-year history of progressive asthenia, macroglossia, dysphonia, and significant weight loss. Despite multiple consultations, including a 2014 neurological exam confirming the presence of highly suggestive bilateral carpal tunnel syndrome (CTS), no clear diagnosis was established. Upon admission, she presented with poor general condition, cachexia, ECOG 2, and typical amyloidosis clinical manifestations including dysphonia, hypophonia, dysphagia, macroglossia, submandibular hypertrophy, periorbital hyperpigmentation, autonomic dysfunction with orthostatic hypotension, constipation, paresthesia, and perimalleolar edema. In contrast to general physical wasting, the patient presented with distinct muscular hypertrophy in the lower limbs. Muscle strength was normal, grade 5/5 Medical Research Council, and deep tendon reflexes were slightly diminished.

Further tests were performed to confirm the high clinical suspicion of systemic amyloidosis and to determine the extent of organ involvement. The involved serum free light chain showed a 2000-fold increase compared to the normal maximum value (9310 mg/L), with a high difference between involved and uninvolved serum free light chains (307 mg/L), with immunoparesis, while the bone marrow smear was consistent with a 9% plasma cell infiltration. Fluorescence in situ hybridization for del17p, t(14;16), or t(4;14) was negative. Renal involvement manifested as nephrotic syndrome, with severe proteinuria (9 g/24 h), and mild creatinine clearance decrease (eGFR>50 mL/min/1.73 m^2^). Although the electrocardiogram and echocardiogram showed no clear amyloidosis-associated changes, NT-proBNP was high (2741 pg/mL), with increased troponin I and troponin T levels. No liver or spleen involvement was observed. Whole-body low-dose CT excluded the presence of osteolytic bone lesions suggestive of multiple myeloma. Abdominal fat pad biopsy confirmed the presence of typical Congo red amyloid deposits with apple-green birefringence under polarized light.

Nerve conduction studies were consistent with bilateral CTS and mild lower limb sensitive polyneuropathy. Needle electromyography described a moderate myopathic pattern in the proximal muscles of the lower limbs, with fibrillation potential.

Subsequently, we confirmed the diagnosis of systemic λAL stage IIIA (European modification of Mayo 2004) with cardiac, renal, soft tissue (musculoskeletal, macroglossia, and CTS) and autonomic nervous system involvement.^[4,5]^

**Figure 1:**
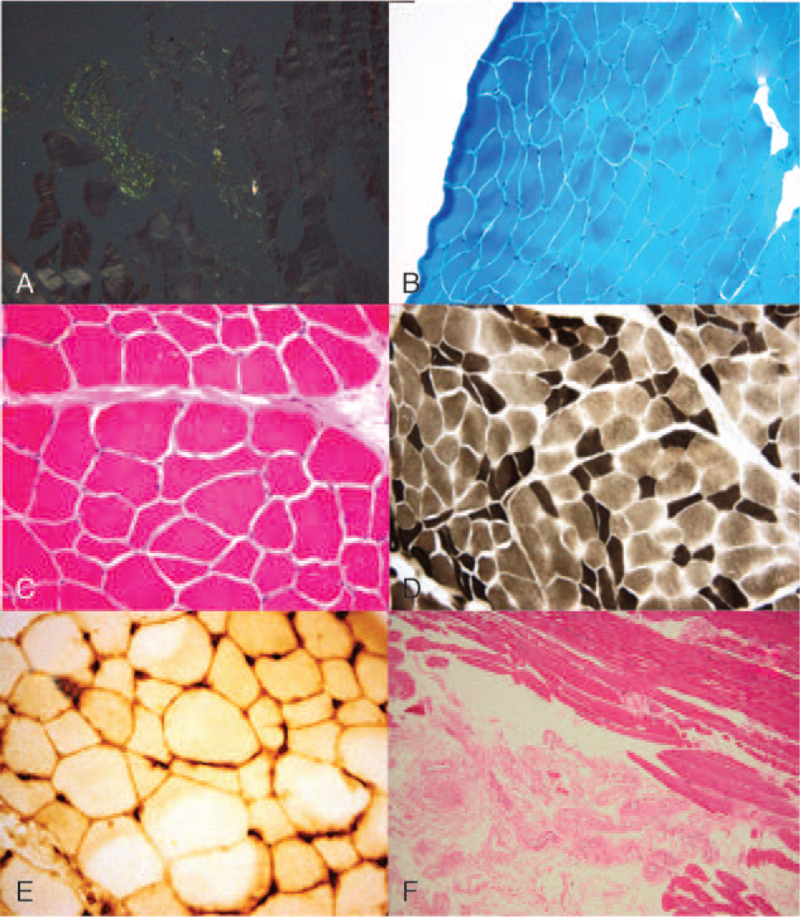
A. Paraffin embedded skeletal muscle, with Congo red staining, showing a characteristic green birefringence under polarized light. **B. and C.** Cryosections of muscle tissue revealing a moderate variability in both size and shape of muscle fibers, with isolated and grouped atrophic fibers elongated/angular or polygonal, both isolated and grouped, but also moderate hypertrophic fibers, modified by Gomori trichrome stain (A), hematoxylin and eosin (B), ×100. **D.** Atrophy mainly affects dark type II muscle fibers (ATP-ase pH 9.4 on cryosections, ×200). **E.** Sarcolemma hypererexpression of MHC class I on muscle tissue cryosections, ×400. **F.** Enlarged muscle interstitium, with thickened vascular walls on paraffin-embedded muscle tissue. HE ×100.

According to existing treatment guidelines for transplant ineligible patients, in September 2017, we initiated the first line of therapy, consisting of bortezomib, cyclophosphamide, and dexamethasone. Despite initial clinical improvement, with partial hematologic (free lambda 1510 mg/L) and renal response (58% reduction of 24H-proteinuria), after 2 cycles of therapy, the patient presented with symptom setback, shortness of breath, accentuated muscular hypertrophy with painful myalgia, fasciculation, and worsening paresthesia. Additional extensive neurological evaluation showed no significant changes compared to baseline, with persistent CTS, mild sensitive polyneuropathy, and electromyographic myopathic changes. Since the cause of the progressive muscular hypertrophy was not entirely clear, an open muscle biopsy of the left vastus lateralis was performed. The initial histopathology report described nonspecific changes, consisting of atrophic and rare hypertrophic fibers associated with mild denervation. Although corticotherapy could have explained the findings, the samples were minutely re-analyzed with additional Congo red staining under polarized light. Surprisingly, apple-green birefringent amyloid deposits, unnoticed on routine staining, were located around the microvessel walls. Immunohistochemical staining revealed sarcolemma hyperexpression of MHC class I. No additional signs of necrosis, mitochondrial abnormalities, fibrosis, rimmed vacuoles, or reducing bodies were observed (Fig. 1).

Considering the clinical deterioration, insufficient hematological response, and progressive muscular hypertrophy, now regarded as a direct sign of additional light chain deposition, a second line of therapy was initiated. The new lenalidomide-based regimen (bortezomib, lenalidomide, and dexamethasone) conferred a deepened hematological response (free lambda 516 mg/L) and clinical improvement. After completing 6 bortezomib, lenalidomide, and dexamethasone cycles, the treatment was continued with lenalidomide maintenance. Unfortunately, after just 2 months, she showed symptomatic disease progression, with a significant increase in free lambda chains (969 mg/L), water retention, dyspnea, increased muscle contracture, and dysphagia.

In May 2018, a third line of therapy was initiated, consisting of the second-generation proteasome inhibitor carfilzomib, associated with the lenalidomide backbone (carfilzomib, lenalidomide, dexamethasone). The new regimen led to a complete hematological response, full resolution of muscle pseudohypertrophy, complete renal response (absent 24H-proteinuria), and cardiac response (decrease in NT-proBNP).

During the most recent follow-up, more than 1 year after therapy completion, the patient maintained a deep, durable, and complete hematological response. Clinically, the patient has persistent macroglossia and periocular hyperpigmentation, but remarkably, a complete resolution of muscular and neurological manifestations. Moreover, nerve conduction studies and needle electromyography were within the normal range.

## 3 Discussion

Systemic AL amyloidosis is widely underdiagnosed because of nonspecific symptoms and lack of routine Congo red staining. Among the various sites that can be infiltrated by abnormal proteins, the muscular system is one of the rarest.^[6]^

The clinical presentation patterns of amyloid myopathy were defined in a case series by Gertz et al 2016^[6]^, Chapin et al in 2005,^[7]^ and Gertz and Kyle in 1996,^[8]^ and were classified by Giuliani et al^[9]^ in 3 distinct patterns: “skeletal pseudohypertrophy” (with particular characteristics consisting in palpable nodules and wood-like consistency, frequently associated with macroglossia), “the atrophic form” (consistent with muscle weakness, occasionally accompanied by atrophy) and the “mixed phenotype”.^[9]^

According to the existing data, our patient matched the skeletal pseudohypertrophy pattern of muscle involvement in AL amyloidosis by presenting with muscle hypertrophy and macroglossia.

An extensive retrospective study conducted by Gertz et al^[6]^ at the Mayo Clinic described weakness, dysphagia, myalgia, and macroglossia as the main clinical manifestations of amyloid myopathy in AL amyloidosis. Accordingly, our patient presented with all of the commonly reported symptoms. A 2-year median diagnosis delay was recorded in both the Mayo Clinic cohort and the case described here. Interestingly, almost half of the Mayo patients who lacked cardiac involvement showed increased troponin T levels, raising the possibility of defining new indicators of muscular involvement in this subset of patients.^[6]^

Even in the presence of clinical suspicion, leading to muscle biopsy, the diagnosis can be overlooked due to the lack of routine incorporation of Congo red staining. The preliminary histopathology report for our patient described unspecific muscular changes secondary to cortisol exposure, and only after thorough re-examination, the specific green-apple birefringent deposits were reported. Similarly, more than one-third of the patients reviewed in the Mayo cohort had a missed histopathological diagnosis.

Gertz et al^[6]^ reported that melphalan-based induction followed by autologous stem cell transplantation improved overall survival. The median overall survival was 32 months, with worse outcomes in patients with cardiac involvement or more than 2 affected organs.^[6]^

Our patient was considered transplant-ineligible and received continuous therapy over the course of 23 months. Three subsequent lines of therapy, consisting of first- and second-generation proteasome inhibitors, as well as the immunomodulatory agent lenalidomide, were administered with slow reduction of pathological lambda chains. A complete hematologic response was obtained after more than 18 months of therapy, followed by an almost complete resolution of organ dysfunction. Fortunately, the lack of massive cardiac involvement offered the necessary time frame for our patient to obtain a deep hematological response.

Despite the adverse prognostic factors including MAYO stage, high difference between involved and uninvolved serum free light chains, immunoparesis, and high proteinuria, the overall survival has not been reached and currently has a progression-free survival of 12 months. Three years after diagnosis, the patient had a complete hematological and renal response, no pseudohypertrophy, normal electrophysiological and electromyographic examination with complete resolution of CTS, and sensitive polyneuropathy.

The consequences of amyloid deposition in muscle structures are intricate and not entirely understood. Electromyographic changes are similar to those found in patients with inflammatory myopathy, consisting of a myopathic pattern associated with normal nerve conduction.^[3,10]^ Functionally, amyloid can directly decrease muscular elasticity, leading to impaired contraction and weakness.^[3,11,12]^ Additionally, perivascular connective tissue infiltration can lead to impaired muscular nutrition and ischemia, leading to atrophy.^[3,13,14]^ Interestingly, some reports have shown that free light chains could have a trophic effect on human myocytes, leading to muscular hypertrophy.^[15]^

Histopathologically, the previously reported changes consist of atrophy affecting predominantly type II fibers, with peri-vascular, interstitial, perimysium, and endomysium amyloid deposits.^[14]^ Similarly, the muscle biopsy in our case showed moderate atrophy, particularly in type II fibers, associated with mild denervation and peri-vascular amyloid deposits.

## 4 Conclusion

In conclusion, despite its rarity, muscle involvement should be considered in patients diagnosed with AL amyloidosis associated with unexplained muscle hypertrophy or weakness associated with macroglossia or elevated troponin T levels in the absence of clear cardiac involvement. The prognosis of these patients is directly influenced by timely diagnosis before irreversible organ damage has already been established.

## Author contributions

**Conceptualization:** Mirela Draghici, Andreea Jercan, Daniel Coriu.

**Data curation:** Mirela Draghici, Andreea Jercan, Sorina Nicoleta Badelita, Ruxandra Maria Irimia, Daniel Coriu.

**Funding acquisition:** Mirela Draghici, Andreea Jercan, Sorina Nicoleta Badelita, Ruxandra Maria Irimia, Alexandra Eugenia Bastian, Camelia Dobrea, Monica Popescu, Daniel Coriu.

**Investigation:** Mirela Draghici, Andreea Jercan, Sorina Nicoleta Badelita, Alexandra Eugenia Bastian, Camelia Dobrea, Monica Popescu.

**Methodology:** Andreea Jercan, Daniel Coriu.

**Project administration:** Daniel Coriu.

**Resources:** Sorina Nicoleta Badelita, Ruxandra Maria Irimia, Alexandra Eugenia Bastian, Camelia Dobrea, Monica Popescu.

**Supervision:** Daniel Coriu.

**Validation:** Daniel Coriu.

**Visualization:** Andreea Jercan, Alexandra Eugenia Bastian, Camelia Dobrea, Daniel Coriu.

**Writing – original draft:** Mirela Draghici, Andreea Jercan.

**Writing – review & editing:** Mirela Draghici, Andreea Jercan, Sorina Nicoleta Badelita, Ruxandra Maria Irimia, Alexandra Eugenia Bastian, Camelia Dobrea, Monica Popescu, Daniel Coriu.
